# Population genetic structure and connectivity of a riparian selfing herb *Caulokaempferia coenobialis* at a fine-scale geographic level in subtropical monsoon forest

**DOI:** 10.1186/s12870-021-03101-7

**Published:** 2021-07-08

**Authors:** Qiong Fu, Jie Deng, Min Chen, Yan Zhong, Guo-Hui Lu, Ying-Qiang Wang

**Affiliations:** 1 Guangdong Provincial Key Laboratory of Biotechnology for Plant Development, School of Life Sciences, South China Normal University, Guangzhou, China; 2 Guangzhou Key Laboratory of Subtropical Biodiversity and Biomonitoring, School of Life Sciences, South China Normal University, Guangzhou, China

**Keywords:** Genetic variation, Mating system, Fine-scale spatial genetic structure, Gene flow, Seed dispersal

## Abstract

**Background:**

Rivers and streams facilitate movement of individuals and their genes across the landscape and are generally recognized as dispersal corridors for riparian plants. Nevertheless, some authors have reported directly contrasting results, which may be attributed to a complex mixture of factors, such as the mating system and dispersal mechanisms of propagules (seed and pollen), that make it difficult to predict the genetic diversity and population structure of riparian species. Here, we investigated a riparian self-fertilizing herb *Caulokaempferia coenobialis*, which does not use anemochory or zoochory for seed dispersal; such studies could contribute to an improved understanding of the effect of rivers or streams on population genetic diversity and structure in riparian plants. Using polymorphic ISSR and cpDNA loci, we studied the effect at a microgeographic scale of different stream systems (a linear stream, a dendritic stream, and complex transverse hydrological system) in subtropical monsoon forest on the genetic structure and connectivity of *C. coenobialis* populations across Dinghu Mountain (DH) and Nankun Mountain (NK).

**Results:**

The results indicate that the most recent haplotypes (DH: H7, H8; NK: h6, h7, h11, h12) are not shared among local populations of *C. coenobialis* within each stream system. Furthermore, downstream local populations do not accumulate genetic diversity, whether in the linear streamside local populations across DH (*H*: 0.091 vs 0.136) or the dendritic streamside local populations across NK (*H*: 0.079 vs 0.112, 0.110). Our results show that the connectivity of local *C. coenobialis* populations across DH and NK can be attributed to historical gene flows, resulting in a lack of spatial genetic structure, despite self-fertilization. Selfing *C. coenobialis* can maintain high genetic diversity (*H* = 0.251; *I* = 0.382) through genetic differentiation (*G*_ST_ = 0.5915; *F*_ST_ = 0.663), which is intensified by local adaptation and neutral mutation and/or genetic drift in local populations at a microgeographic scale.

**Conclusion:**

We suggest that streams are not acting as corridors for dispersal of *C. coenobialis*, and conservation strategies for maintaining genetic diversity of selfing species should be focused on the protection of all habitat types, especially isolated fragments in ecosystem processes.

**Supplementary Information:**

The online version contains supplementary material available at 10.1186/s12870-021-03101-7.

## Background

Genetic variation in a plant species is determined by mating system, natural selection, evolutionary history, life-history characteristics, and mechanisms of gene flow (dispersal ability of pollen and seed), and these factors can lead to complex genetic structuring of populations within the species [1, 2]. Therefore, the genetic diversity and structure of plant populations can reveal useful information about, and is regarded as the strategic mainstay of, biodiversity and the diversity of a species within and among wild populations inhabiting an ecosystem [3–5]. The increase in human-mediated disturbance in recent decades, such as the destruction of forests and fragmentation of the habitats of many plant species, has led to a rapid loss of biodiversity and consequent changes in the structure of landscapes [6–8]. Thus, an increased understanding of genetic variation and connectivity in species found in habitats of high natural value is key to the development of conservation strategies for small and isolated populations [9, 10]. Forests support about 65% of the world’s terrestrial taxa and have the highest species diversity for many taxonomic groups; thus conserving forest biodiversity is a critical task and has rightly become a key component of many national and international forest management agreements [11]. So far, most studies on the genetic variation and population genetic structure of forest plants have focused on woody plants [12–20], while empirical studies on undergrowth herbs are rare [21–26]. Among the latter, studies on riparian plants in the undergrowth (e.g. *Primula sieboldii*, [22]; *Heliconia metallica*, [27]), are rarer still.

Rivers or streams facilitate movement of individuals and genes across the landscape and are generally recognized as corridors for riparian plants [5, 28]. The passive movement of propagules by water (hydrochory) is an important mode of dispersal for riparian plant species, and it has a significant influence on the composition of riparian plant communities, often promoting species richness [18, 29, 30]. The effectiveness of water as a dispersal vector means that hydrochory is also responsible for high levels of gene flow and thus reduces genetic differentiation between populations as well as greater genetic diversity of downstream populations [31–35]. However, some authors report the opposite results, i.e. that both marked genetic differentiation and genetic discontinuity can be found among populations within a river, suggesting that rivers do not act as corridors for dispersal [36–41]. The above contrary results may be attributed to a number of competing factors (e.g. river/stream, mountains, and fragmentation) that might all be involved to some degree in the historical or current dispersal of seeds or vegetative propagules, leading to difficulty in predicting the genetic diversity and population structure of riparian species [41]. Mountain ridges act as geographical barriers and can hinder gene flow, causing genetic differentiation in plants [42], and habitat fragmentation may disrupt or reduce gene flow and erode genetic variation in plants [43]. Moreover, the breeding system and dispersal ability of pollen and seed, which are the most important factors affecting the spatial genetic structure and dynamics of populations within species [44–46], have not been considered in previous studies on riparian plants. In outcrossing plants, pollen dispersal has the potential for long-distance gene transport by wind or animal pollinators, and thus is generally an important component of total gene flow [47–49]. Previous studies have shown that outcrossing plants can preserve a degree of genetic diversity through frequent gene flow among populations, while genetic differentiation can effectively be eliminated when gene flow per generation is very low [50, 51]. In contrast, the movement of seeds is the main component of gene flow for selfing plants due to lack of pollen migration among demes of selfing species. Numerous studies on genetic variation have shown that within-population diversity is typically reduced in selfing species relative to outcrossing species, but genetic differentiation among populations is strengthened [52–56]. In fact, the movement of seeds may influence the spatial distribution of genetic diversity and favors genetic connectivity between populations of both selfing and outcrossing plants. Most seeds disperse very close to the source plants, and thus spatial aggregation of seeds with shared lineages is expected in most situations [57]. However, there are several factors, such as hydrochory, anemochory and zoochory, that may influence secondary seed dispersal and alter seed distribution patterns [58–61], which accordingly may influence the spatial distribution of genetic diversity and genetic connectivity between populations. As a result, because of this complex mixture of factors, it can be difficult to fully understand what drives genetic diversity and population structure in riparian plants. Thus, studies of a species with no pollen migration (selfing) and without anemochory or zoochory seed dispersal are needed; such studies could contribute to an improved understanding of the effect of rivers or streams on population genetic diversity and structure in riparian plants. In addition, numerous previous studies on riparian plants were carried out on a macrogeographic scale, with few performed at a fine-scale geographic level (e.g. *Hibiscus moscheutos*, [62]; *Primula sieboldii*, [22]; *Mauritia flexuosa*, [17]). However, knowledge of the extent to which rivers or streams impact fine-scale population genetic patterns and, in particular, how factors such as the direction of river flow structure populations, is essential for understanding the likely effect of habitat on riparian plants.

*Caulokaempferia coenobialis* (Hance) K. Larsen (Fig. S1) is a deciduous perennial herb that grows on steep cliffs along streams in shady, humid monsoon forests in south China [63, 64]. The plant is nonclonal and self-fertilizing by sliding pollen [64, 65] and seeds disperse by rain splash [65–67]. Therefore, selfing *C. coenobialis* represents a good system with which to investigate the effect of rivers or streams on population genetic diversity and structure in riparian plants, because it can disentangle the relative influences on the genetic structure of pollen migration and secondary seed dispersal by anemochory or zoochory. In this study, we assess the levels of genetic variation and differentiation within and between local populations of selfing *C. coenobialis* on a microgeographic scale using cpDNA and ISSR (inter-simple sequence repeat) data, focusing on the following questions: (1) does selfing *C. coenobialis* show low genetic diversity and significant spatial genetic structure on a microgeographic scale, as theory predicts? (2) do streams facilitate the dispersal of *C. coenobialis* seeds among local populations along the longitudinal course of a stream, thus leading to an accumulation of genetic diversity in downstream local populations, as theory predicts?

## Results

### ISSR, cpDNA characteristics and genetic diversity

**Fig. 1:**
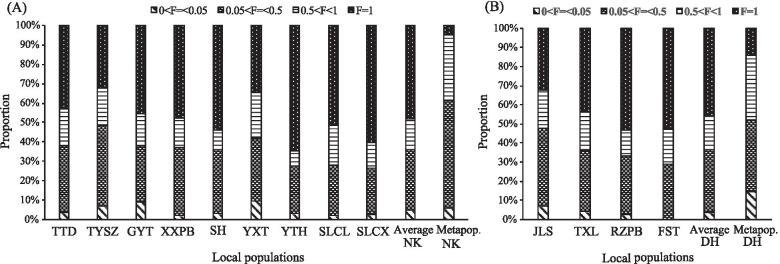
Distribution of ISSR gene frequency in local populations within metapopulations NK (**A**) and DH (**B**) of *Caulokaempferia coenobialis*

The ten ISSR primers produced 218 reproducible bands (an average of 21.8 bands per primer) from the 13 local populations in metapopulations DH and NK, of which 213 (97.7%) were polymorphic and 6 (2.8%) were specific (Table 1, Table 2). At the metapopulation level, the averages of Nei’s genetic diversity (*H*) and the Shannon indices (*I*) of *C. coenobialis* were 0.251 and 0.382, respectively. The genetic diversity index of NK was greater than that of DH (*H*: 0.298 vs 0.204; *I*: 0.450 vs 0.313). At the local population level, genetic diversity index ranged from 0.058 to 0.136 (*H*, average of 0.098) and 0.089 to 0.211(*I*, average of 0.152). Local populations JLS (DH) and TYSZ (NK) showed the highest levels of genetic diversity (*H* = 0.136, *I* = 0.209; *H* = 0.136, *I* = 0.211, respectively), while local populations FST (DH; *H* = 0.091, *I* = 0.138) and SLCX (NK; *H* = 0.058, *I* = 0.089) exhibited the lowest levels. Among all local populations except JLS (DH) and TYSZ (NK), common loci (i.e. found in all individuals/local population: gene frequency = 100%) accounted for the highest proportion of amplified fragments (34.3–60.4%), while low-medium gene frequency loci (5% < gene frequency ≤ 50%) accounted for a higher proportion of amplified fragments (23.6–41.9%) than medium–high gene frequency loci (50% < gene frequency < 100%) (13.2–24.1%) (Fig. 1). Rare loci (gene frequency ≤ 5%) represented a very low proportion in all 13 local populations (0.0–9.5%). However, at the metapopulation level, both common loci and rare loci of DH and NK were less prevalent, i.e. 14.2% and 14.8%, and 4.8% and 5.8%, respectively, but loci with low-medium and medium–high gene frequency accounted for a higher proportion of amplified fragments, i.e. 37.2% and 33.9%, and 55.8% and 33.7%, respectively.

**Table 1:** Location and genetic diversity parameters based on cpDNA and ISSR data in *Caulokaempferia coenobialis* local populations

Metapopulation /local population	Latitude (°N)	Longitude (°E)	Altitude (m)	*n* (cpDNA/ISSR)	cpDNA			ISSR (Fis = 1)					
					*h*	π × 10^–3^	Haplotypes (no. of individuals)	*H*	*I*	PL	PPL (%)	NL	NS
Metapop. DH													
Local pop													
JLS	23.1784	112.5027	600	16/20	0.733	1.420	H1(7), H2(3), H3(3), H4(2)	0.136	0.209	98	44.95	145	2
TXL	23.1788	112.5246	477	5/20	0.700	3.170	H3(1), H7(3), H8(1)	0.095	0.150	77	35.32	137	1
RZPB	23.1792	112.5293	450	5/20	0.800	1.110	H2(1), H5(2), H6(2)	0.093	0.142	63	28.90	135	0
FST	23.1758	112.5381	400	5/10	0.400	0.630	H2(4), H6(1)	0.091	0.138	60	27.52	127	0
Average					0.658	1.583		0.104	0.160	74.5	34.17	136	0.75
Total					0.855	2.790		0.204	0.313	157	72.0	183	3
Metapop. NK													
Local pop													
TTD	23.6439	113.8455	571	5/20	0.600	1.010	h3(3), h9(2)	0.112	0.176	85	38.99	149	0
TYSZ	23.6384	113.8610	336	10/20	0.327	0.280	h1(2), h3(9)	0.136	0.211	100	45.87	148	0
GYT	23.6325	113.8555	350	5/20	0.400	0.340	h6(4), h7(1)	0.110	0.172	88	40.37	162	0
XXPB	23.6311	113.8668	307	5/20	0.000	0.000	h8(5)	0.109	0.167	76	34.86	145	0
SH	23.6402	113.8911	340	5/20	0.000	0.000	h4(5)	0.079	0.121	54	24.77	117	0
YXT	23.6113	113.8551	550	5/20	0.600	0.500	h1(3), h5(2)	0.106	0.164	90	41.28	137	2
YTH	23.6105	113.8697	715	5/20	0.400	0.340	h5(1), h12(4)	0.066	0.101	46	21.10	130	0
SLCL	23.6104	113.8772	480	5/20	0.600	1.010	h1(3), h2(2)	0.083	0.131	66	30.28	136	1
SLCX	23.6092	113.8753	420	5/20	0.800	1.180	h1(2), h10(2), h11(1)	0.058	0.089	42	19.27	106	0
Average					0.414	0.518		0.095	0.148	71.9	32.98	136.7	0.33
Total					0.883	1.960		0.298	0.450	198	90.83	208	3
Mean													
Local pop					0.489	0.845		0.098	0.152	72.7	33.3	136.5	0.46
Metapop					0.869	2.375		0.251	0.382	177.5	81.42	195.5	3

*n* Sample sizes for cpDNA/ISSR analyses, *h* Chlorotype diversity, π Nucleotide diversity, *H *Nei’s gene diversity, *I *Shannon’s information index, *PL* Number of polymorphic loci, *PPL* Percentage of polymorphic loci, *NL* number of loci, *NS* Number of specific bands, *DH* Dinghu Mountain, *NK* Nankun Mountain

**Table 2:** Attributes of two cpDNA and ten ISSR primers used in the present study

Primer	Sequence 5´to 3´	*T*/°C Annealing temperature	SR	NT	NP
cpDNA					
trnL intron	F: CGAAATCGGTAGACGCTACG	48			
	R: GGGGATAGAGGGACTTGAAC				
psbJ-petA	F: ATAGGTACTGTAR*CYGGTATT	52			
	R: AACAR*TTY*GAR*AAGGTTCAATT				
ISSR					
807	(AG)_8_ T	52	230–1500	21	20
808	(AG)_8_C	52	180–1700	20	19
810	(GA)_8_ T	52	210–1520	22	22
835	(AG)_8_Y*C	58	170–1700	19	17
836	(AG)_8_Y*A	50	250–1700	20	20
840	(GA)_8_Y*T	52	200–1500	22	22
841	(GA)_8_Y*C	52	180–1780	23	22
847	(CA)_8_R*C	52	280–1700	24	24
859	(TG)_8_RC	49	290–1700	24	24
887	DVD*(TC)_7_	52	220–1700	23	23
Total	-	-	-	218	213

^*^Y: C/G, R: A/T, D: A/G/T; *SR* Size range of amplified fragments, *NT* Number of total bands, *NP* Number of polymorphic bands

**Fig. 2:**
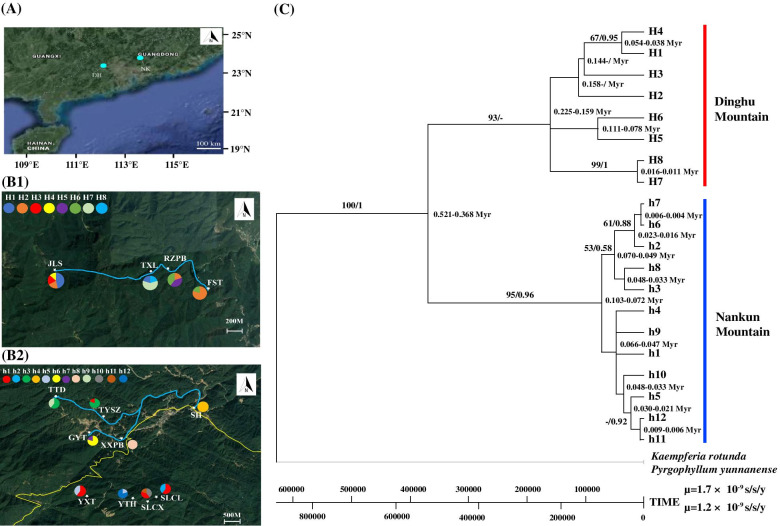
(A) Location of metapopulations DH and NK; (B) Geographic distribution of the chloroplast (cp) DNA haplotypes of *Caulokaempferia coenobialis* detected in local populations within metapopulations DH (1) and NK (2). Light blue and yellow lines indicate streams and G355 National Road, respectively; (C) Maximum likelihood (ML) tree of cpDNA haplotypes of *C. coenobialis*. Bootstrap values (%) based on ML analysis and posterior probability values (BS/PP) are indicated above/below the branches and the coalescence time for each lineage is indicated at nodes. The original satellite imagery was obtained from Google Map (Map data ©2019 Google; https://maps.google.com/) and modified with Adobe Illustrator CS6 (Adobe Systems Incorporated, San Jose, CA, USA)

The concatenated and aligned cpDNA sequences for metapopulations DH and NK were a total of 1262 and 1191 base pairs (bp) long, respectively. The polymorphisms identified 20 haplotypes in DH and NK, of which eight haplotypes (H1-H8) resided in DH and 12 haplotypes (h1-h12) resided in NK (Table 1, Fig. 2). At the metapopulation level, cpDNA data revealed high estimates of the average chlorotype diversity (*h* = 0.869) and average nucleotide diversity (π = 2.375 × 10^–3^). At the local population level, haplotype diversity and nucleotide diversity ranged from 0 to 0.800 (average 0.489) and from 0 to 3.170 × 10^–3^ (average 0.845 × 10^–3^), respectively. All local populations except SH and XXPB (NK) were polymorphic, while local populations JLS (DH) and SLCX (NK) contained four and three different haplotypes, respectively.

### Genetic differentiation and gene flow

Based on the ISSR dataset (Table 3), the genetic differentiation value (*G*_ST_) for the local populations of *C. coenobialis* in DH and NK was 0.5034 and 0.6796, respectively, which indicates that 49.66% and 32.04% of total genetic variability was distributed within local populations in DH and NK, respectively. The estimated gene flow (*N*m) per generation among local populations in DH and NK was 0.4933 and 0.2357, respectively. In NK, the genetic differentiation among the transverse hydrological system local populations in NK-II were greater than among the dendritic streamside system local populations in NK-I (*G*_ST_: 0.6589 vs 0.5982); accordingly, the mean estimated gene flow (*N*m) among local populations in NK-II is lower than that in NK-I (0.2588 vs 0.3359). AMOVA results (Table 4) were consistent with the Nei’s genetic differentiation statistics, showing that 53.00% and 69.00% of the total variation was partitioned among local populations of DH and NK, respectively. In NK, 63.00% and 71.00% of the total variation was partitioned among local populations in NK-I and NK-II, respectively.

**Table 3:** Statistics for genetic differentiation among local populations of *Caulokaempferia coenobialis* based on cpDNA and ISSR data (*F*is = 1)

Metapopulation	cpDNA				ISSR			
	*H*_T_	*H*s	*F*_ST_	*N*m	*H*_T_	*H*s	*G*_ST_	*N*m
Dinghu Mountain	0.920 (0.0672)	0.658 (0.0886)	0.556 (NC)	0.40	0.2088	0.1037	0.5034	0.4933
Nankun Mountain								
All populations	0.931 (0.0332)	0.400 (0.0943)	0.770 (0.0981)	0.14	0.2978	0.0954	0.6796	0.2357
NK-I	0.896 (0.0730)	0.267 (0.1229)	0.863 (0.0991)	0.07	0.2719	0.1092	0.5982	0.3359
NK-II	0.847 (0.0532)	0.600 (0.0816)	0.477 (0.1552)	0.55	0.2291	0.0781	0.6589	0.2588

*H*_T_ Total local population diversity, *H*_S_ Average within local population diversity, *F*_ST_ and *G*_ST_ Coefficient of gene differentiation among local populations, *N*m Estimate of gene flow from *G*_ST_, *NK-I* Dendritic streamside system local populations in Nankun Mountain, *NK-II* Transverse hydrological system local populations in Nankun Mountain

**Table 4:** The analysis of molecular variance (AMOVA) for cpDNA data and ISSR data among local populations of *Caulokaempferia coenobialis*

Metapopulation	Source of variation	cpDNA					ISSR				
		d.f	SS	VC	variance (%)	*R*_ST_	d.f	SS	Est. var	variance (%)	*Ф*_ST_
Dinghu Mountain	Among local populations	3	25.967	1.1545	54.63	0.546	3	747.207	13.817	53.00	0.531
	Within local populations	26	24.933	0.9590	45.37		66	805.450	12.204	47.00	
	Total	29	50.900	2.1135	100		69	1552.657	26.021	100	
Nankun Mountain											
All populations	Among local populations	7	41.659	1.1272	84.55	0.846	8	3971.111	24.272	69.00	0.689
	Within local populations	33	6.800	0.2061	15.45		171	1872.100	10.948	31.00	
	Total	40	48.659	1.3333	100		179	5843.211	35.220	100	
NK-I	Among local populations	5	29.987	1.1279	86.50	0.865	4	1772.600	21.531	63.00	0.632
	Within local populations	25	4.400	0.1760	13.50		95	1190.800	12.535	37.00	
	Total	30	34.387	1.3039	100		99	2963.400	34.066	100	
NK-II	Among local populations	3	7.500	0.4100	47.67	0.477	3	1316.050	21.486	71.00	0.706
	Within local populations	16	7.200	0.4500	52.33		76	681.300	8.964	29.00	
	Total	19	14.700	0.8600	100		79	1997.350	30.450	100	

*d.f.* Degrees of freedom, *SS* Sum of squares, *VC* Variance components, *Est. var.* Estimated variance, *R*_ST_*/Ф*_ST_ Statistics analogous to *F*_ST_ statistics; all levels of variation were significant, *NK-I* Dendritic streamside system local populations in Nankun Mountain, *NK-II* Transverse hydrological system local populations in Nankun Mountain

For cpDNA, the coefficient of genetic differentiation (*F*_ST_) among local populations was estimated as 0.556 and 0.770 for DH and NK, respectively (Table 3). The *N*m per generation among local populations in DH and NK was 0.40 and 0.14, respectively. In NK, the *F*_ST_ among NK-II local populations was lower than that among NK-I local populations (0.477 vs 0.863). AMOVA (Table 4) revealed that the molecular variance was partitioned among local populations both in DH (54.63%) and NK (84.55%). However, 86.5% of the total molecular variance resided among local populations in NK-I, while 47.67% of the total molecular variance was attributable to the divergence among local populations in NK-II.

### Relationships of cpDNA haplotypes

In total, 20 cpDNA haplotypes were identified in DH and NK, and of these 14 were private haplotypes restricted to a single local population. Six haplotypes were shared among local populations, of which H1, H2 and H6 were shared among DH local populations, and h1, h3 and h5 were shared among NK local populations (Table 1, Fig. 2 B1, B2). Of the latter, h1 was shared between NK-I and NK-II, while h3 and h5 only occurred in NK-I and NK-II, respectively (Fig. 2 B2).

The ML chronogram derived from cpDNA of *C. coenobialis* local populations in DH and NK supported the 20 haplotypes as a monophyletic group with a bootstrap value of 100%, with an estimated crown age of ca. 0.521–0.368 Myr (Fig. 2 C). This monophyletic group was further split into two main lineages: the NK lineage (ML bootstrap support (BS) = 95%, PP = 0.96) and the DH lineage (ML BS = 93%, PP = -). The most recent common ancestor of the DH lineage (0.225–0.159 Myr) existed earlier than that of the NK lineage (0.103–0.072 Myr). The divergence time estimates (ca. 0.016–0.011 Myr) suggest that H7 and H8 were the most recent haplotypes to arise in DH. The h6, h7, h11 and h12 haplotypes arose most recently in NK, with divergence times of ca. 0.009–0.004 Myr.

### Genetic clustering and spatial genetic structure within metapopulations

**Fig. 3:**
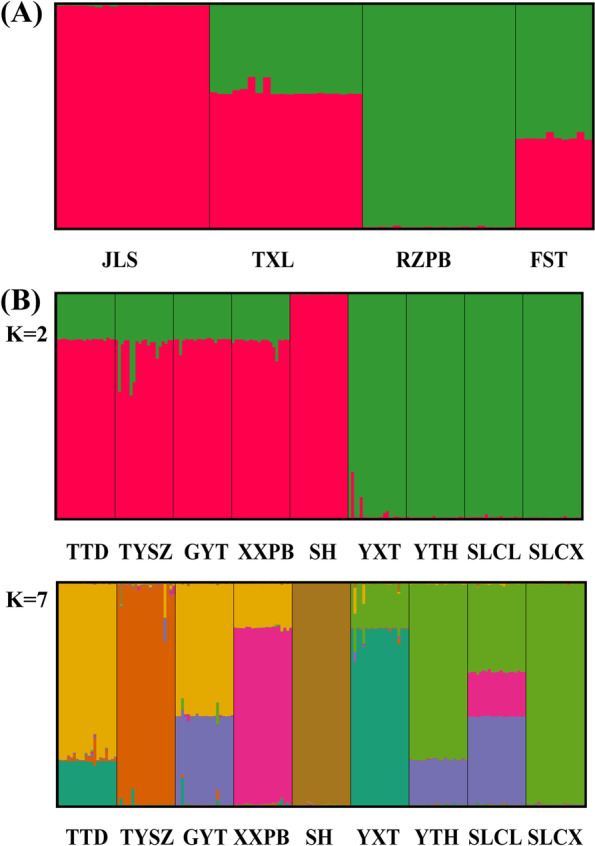
Genetic-group structure shown by STRUCTURE analysis based on ISSR data for metapopulations DH (**A**, K = 2) and NK (**B**, K = 2, 7) of *Caulokaempferia coenobialis*. Each individual vertical bar represents an individual plant and the black vertical bars separate the local populations, while different colors represent different gene pools

Bayesian genetic STRUCTURE analyses revealed that the log likelihood reached a maximum value at K = 2 and assigned all local populations to two genetic clusters in DH (Fig. 3 A; Fig. S2 A). In local populations JLS and RZPB, all individuals within each local population were assigned to the same genetic clusters. However, there was a high degree of admixture of two gene pools in all individuals in local populations TXL and FST. In NK, with K = 2 (the second highest ΔK value), all local populations were assigned to two genetic clusters (Fig. 3 B; Fig. S2 B), in which almost all individuals were assigned to the same genetic cluster within local populations except for four local populations of NK-I (TTD, TYSZ, GYT, XXPB); this suggests a higher level of admixture of the two gene pools within these four local populations. At the highest log likelihood (K = 7), all local populations in NK could be assigned to seven genetic clusters (Fig. 3 B). Except for three local populations (TYSZ, SH and SLCX), all individuals within each local population showed signs of genetic admixture.

**Fig. 4:**
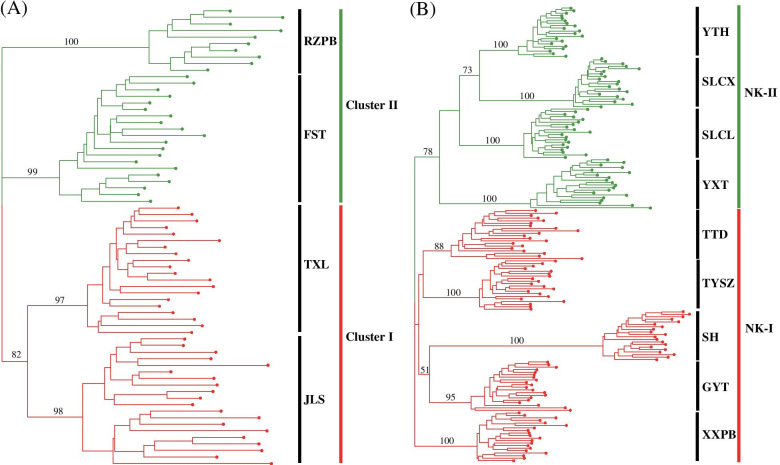
Unrooted neighbor-joining (NJ) tree based on ISSR Nei’s genetic distance for individuals of *Caulokaempferia coenobialis* within metapopulations DH (**A**) and NK (**B**). Bootstrap values (> 50%) are indicated above the branches

**Fig. 5:**
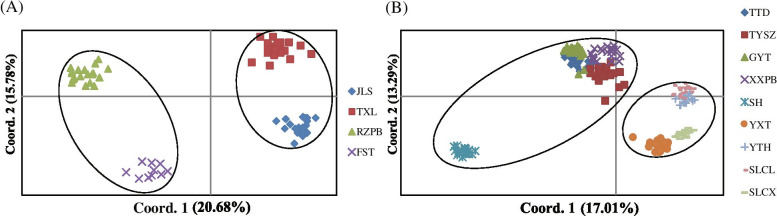
Scatterplot of the principal coordinate analysis (PCoA) based on 218 ISSR genotypes from all sampled individuals in local populations of *Caulokaempferia coenobialis* in metapopulations DH (**A**) and NK (**B**). Different colors represent different local populations

All individuals from the same local populations of *C. coenobialis* in DH and NK were clustered together in the unrooted neighbor-joining (NJ) trees based on ISSR Nei’s genetic distance (Fig. 4). In DH, four local populations were classified into two clusters with a similarity index value of 820/1,000 (Fig. 4 A), which comprised two upstream local populations (cluster I: JLS and TXL; highlighted red) and two downstream local populations (cluster II: RZPB and FST; highlighted green), respectively. In NK, nine local populations were grouped into two clusters with a similarity index value of 780/1,000 (Fig. 4 B), of which one cluster (cluster NK-I; highlighted red) comprised the five dendritic streamside system local populations, while the other (cluster NK-II; highlighted green) comprised the four transverse hydrological system local populations, respectively. Cluster NK-I could be divided into two further groups with three clades. One group consisted only of all individuals of local population XXPB, and the other group consisted of two clades with low bootstrap values, in which local populations GYT and SH formed one clade, and local populations TYSZ and TTD formed the other clade. Similarly, cluster NK-II also formed two groups with three well-resolved clades, of which one group comprised all individuals from local population YXT. The other group consisted of two branches, local population SLCL and another clade including local populations YTH and SLCX. The UPGMA dendrogram (Fig. S3) based on Nei's similarity coefficient also showed that all individuals from the same local populations in DH and NK clustered together. However, the four local populations in DH were classified into two clusters with three well-resolved clades (Figure S3 A), a result different from that in the NJ tree. In addition, the nine local populations in NK were separated into two clusters with four clades in the UPGMA dendrogram with a similarity index value of 0.54 (Figure S3 B), a result conflicting with the NJ tree. The PCoA analysis (Fig. 5) revealed a pattern that was broadly consistent with the unrooted NJ tree and the genetic-group structure, in which the local populations of *C. coenobialis* in both DH and NK were classified into two clusters, respectively, and all individuals from the same local populations were clustered together.

**Fig. 6:**
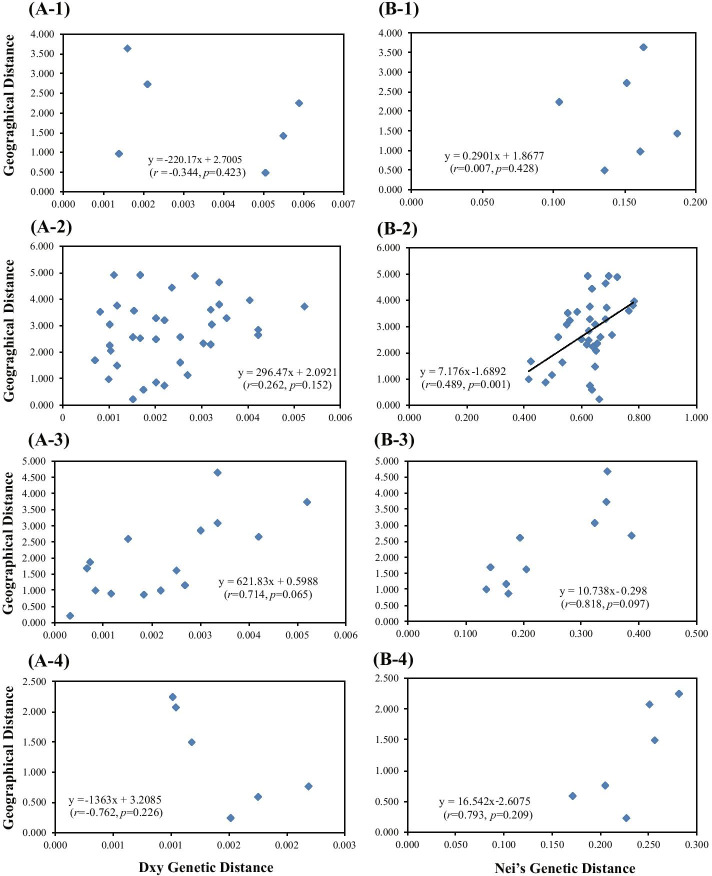
Plot of Mantel test based on cpDNA data (A) and ISSR data (B) showing the relationships of genetic and geographic distances in local populations of *Caulokaempferia coenobialis* from metapopulations (1) DH and (2) NK, as well as the two subsets (3) NK-I and (4) NK-II in NK

**Fig. 7:**
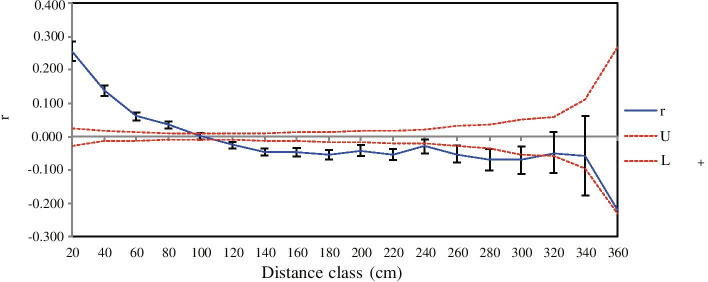
Correlogram showing the spatial autocorrelation coefficient r within the local population TXL in metapopulation DH of *Caulokaempferia coenobialis*. U and L represent the 95% two-tailed confidence interval, which was calculated based on 999 permutations

The Mantel test showed that there was no significant isolation-by-distance relationship across local populations in DH based on both cpDNA (*r* = -0.344, *p* = 0.423) and ISSR (*r* = 0.007, *p* = 0.428) data (Fig. 6 A-1, B-1). However, the genetic divergence of all local populations in NK based on ISSR data was weakly correlated with geographic distance (*r* = 0.489, *p* = 0.001) (Fig. 6 B-2), although no such correlation was observed using cpDNA data (*r* = 0.262, *p* = 0.152) (Fig. 6 A-2). In addition, there was no correlation between genetic and geographical distances among local populations of *C. coenobialis* from either NK-I or NK-II based on cpDNA (*r* = 0.714, *p* = 0.065; *r* = -0.762, *p* = 0.226, respectively) or ISSR (*r* = 0.818, *p* = 0.097;* r* = 0.793, *p* = 0.209, respectively) data (Fig. 6 A, B). Spatial autocorrelation analysis indicated that significant positive spatial genetic structure was detected at 20–92 cm (Fig. 7).

## Discussion

### Can selfing *C. coenobialis* maintain high genetic diversity on a microgeographic scale?

The mating system is a major factor affecting the genetic variability of plant species. Numerous studies have shown that selfing plant species have less genetic diversity at both the population and species levels than outcrossing species [52, 68–70]. In contrast, comparative studies on two closely related selfing and outcrossing *Zingiber* species at both the macrogeographic and microgeographic levels [55, 56] revealed that, although the level of population/subpopulation genetic diversity in selfing *Z. corallinum* was significantly lower than that in outcrossing *Z. nudicarpum*, the level of species/metapopulation genetic diversity of selfing *Z. corallinum* was comparable to that of outcrossing *Z. nudicarpum*. Our results are consistent with those of the above comparative study [56], i.e. the level of metapopulation genetic diversity in selfing *C. coenobialis* is comparable to that of outcrossing *Z. nudicarpum* (*H* = 0.251 vs 0.2246, *p* = 0.730; *I* = 0.382 vs 0.3480, *p* = 0.776) despite the within-population genetic diversity (Nei’s *H* = 0.098) being significantly lower than that in outcrossing *Z. nudicarpum* (*H* = 0.098 vs 0.1464, *p* = 0.003; *I* = 0.152 vs 0.2257, *p* = 0.004). This implies that, similarly to outcrossing species, selfing plant species can maintain a high level of species genetic diversity—albeit by using different strategies—on both the microgeographic and macrogeographic scales.

Unlike outcrossing species, the high species genetic diversity level of selfing plant species may result from a low pollen migration rate, which leads to high levels of genetic differentiation among populations [71, 72]. Without migration among demes of local populations in selfing *C. coenobialis* metapopulations, any mutation that arises in a particular local population may fix in that local population and cannot spread to other local populations. The present results based on ISSR and cpDNA data confirmed that the genetic differentiation among local populations was relatively high in both DH (*G*_ST_ = 0.5034, *F*_ST_ = 0.556) and NK (*G*_ST_ = 0.6796, *F*_ST_ = 0.770) metapopulations. The proportion of ISSR common loci was highest within local populations of *C. coenobialis*, but common loci and low-to-medium gene frequency loci accounted for the lowest proportion and the highest proportion within metapopulations DH and NK, respectively (Fig. 1). Together, these results indicate that allelic loci vary substantially among local populations, thus leading to a high level of genetic diversity in selfing *C. coenobialis* on a microgeographic scale (metapopulations DH and NK). Evolutionary theory predicts that genetic drift will result in substantial local differentiation if *N*m < 1 [73, 74], since gene flow between populations is limited by the extent of pollen and seed dispersal and is insufficient to counter the effects of random drift [2, 75]. This is the case for selfing *C. coenobialis* in our study. The estimates of ISSR gene flow (*N*m) between local populations of *C. coenobialis* in DH and NK were only 0.4933 and 0.2357 on average (*N*m < 1), respectively, which can be attributed to a lack of pollen migration within and among local populations [64, 65], as well as to restricted seed dispersal [66, 67], as confirmed by a significant positive autocorrelation of spatial genetic structure being detected at only 0.20—0.92 m within local populations (Fig. 7). In addition, the large numbers of private cpDNA haplotypes that are derived from ancestral haplotypes (DH: H3, H4, H5, H7, H8; NK: h2, h4, h6-h12) reside in local populations, indicating that the local populations of *C. coenobialis* are highly heterogeneous, thus causing an increase in genetic variation [76]. Based on the above, we suggest that selfing *C. coenobialis* can maintain high genetic diversity through differentiation intensified by local adaptation and neutral mutation and/or by the stochastic force of genetic drift in local populations on a microgeographic scale. Thus, conservation strategies for maintaining and improving genetic diversity in selfing species should be focused on the protection of all habitat types, especially isolated fragments in ecosystem processes.

### Do local populations of *C*. *coenobialis* show significant spatial genetic structure on a microgeographic scale, like most selfing populations?

Self-fertilizing species, with their reduced genetically effective population size and gene flow processes, should be characterized by strong spatial genetic structure [52, 77–79]. Contrary to expectations, the Mantel tests based on both ISSR and cpDNA data suggested no significant correlation between geographical and genetic distance (ISSR: *r* = 0.007, *p* = 0.428; cpDNA: *r* = -0.344, *p* = 0.423) among local populations of the DH metapopulation, implying that historical dispersal and/or contemporary gene flow by seed dispersal and pollen movement might occur among local populations [75, 80]. Due to the self-fertilization mechanism of *C. coenobialis* and thus lack of pollen migration within and among local populations, together with limited seed dispersal, which was confirmed by the low gene flow (*N*m = 0.4933) between local populations of *C. coenobialis* across DH, contemporary gene flow should not occur among local populations. Moreover, our results showed that some ancestral haplotypes (H1, H2, and H6) were shared in DH local populations, but not the most recent haplotypes (H7, H8). This confirmed that historical dispersal, but not contemporary gene flow, has occurred among local populations of *C. coenobialis* across DH.

Similarly, Mantel tests based on cpDNA data showed that there was no significant spatial genetic structure in local populations across NK (*r* = 0.262, *p* = 0.152) and that some ancestral haplotypes (h1, h3, h5) were shared among local populations. These results also indicate that historical dispersal has occurred in local populations across NK. However, a weak relationship between local populations was detected in the ISSR data (*r* = 0.489, *p* = 0.001) and the gene flow was very low (*N*m = 0.2357), implying that contemporary gene flow between local populations is limited by restricted seed dispersal and absence of pollen movement [2, 75]. This is confirmed by the lack of pollen migration within and among local populations of *C. coenobialis* [64, 65] and the limited seed dispersal [66, 67]. Our results also showed a lack of the most recent shared haplotypes (h6, h7, h11, h12) among local populations. Together, these results show that local populations of *C. coenobialis* across NK may have undergone shrinking and fragmentation relatively recently, and that this was accompanied by a greatly decreased gene flow and increased genetic differentiation among local populations. Numerous studies have shown that fragmentation decreases habitat size and increases habitat isolation of populations in many terrestrial ecosystems, and that this reduces gene flow and genetic variation, while increasing the inter-population genetic divergence of plant populations [81–86]. The present results based on ISSR and cpDNA data also confirmed that genetic differentiation among local populations of *C. coenobialis* across NK was substantial. In fact, as a result of increasing tourism in the NK region, habitat connectivity among local populations of *C. coenobialis* has been destroyed by roads, farm lands and holiday villages, which have likely hindered propagule dispersal. The cluster and STRUCTURE results reveal a clear pattern of population structure in *C. coenobialis*, with two clusters corresponding to two areas in NK, the West-North area (cluster NK-I: the dendritic streamside system local populations TTD, TYSZ, GYT, XXPB and SH) and the East-South area (cluster NK-II: the transverse hydrological system local populations YXT, YTH, SLCL and SLCX). The two areas are separated by the G355 National Road along a valley. Mantel tests based on cpDNA and ISSR data applied to the two clusters separately suggest that there is no significant correlation between geographical and genetic distance in both the dendritic streamside system local populations (ISSR: *r* = 0.818, *p* = 0.097; cpDNA: *r* = 0.714, *p* = 0.065) and the transverse hydrological system local populations (ISSR: *r* = 0.793, *p* = 0.209; cpDNA: *r* = 0.762, *p* = 0.226). This is also consistent with historical dispersal (historical gene flows), but not recent gene flow.

From all the above, despite self-fertilization in *C. coenobialis*, we know that the connectivity of local populations across both DH and NK can be attributed to historical gene flows, resulting in a lack of spatial genetic structure on a microgeographic scale. Our results also showed that the shared haplotypes all arose before 0.0158 Myr, between the Holocene and Pleistocene, implying that the historical gene flows among local populations of *C. coenobialis* might have happened before the Holocene in DH and NK, namely in the Pleistocene, and are likely attributable to the influence of neotectonic activity in this era. Geological studies [87, 88] have shown that, as a result of neotectonic activity in the Pleistocene, the region of the Earth’s crust that included Dinghu Mountain and Nankun Mountain was lifted up intermittently, and that this was accompanied by the formation of steep terrain and deep-cut valleys in mountainous areas, the exposure of the bedrock in the valley floor, and the development of complex hydrological systems.

### Does secondary seed dispersal of *C. coenobialis* occur along the longitudinal course of a stream, thus leading to accumulation of genetic diversity in downstream local populations?

Studies have shown that gene flow between populations is largely congruent with river basins and the direction of water flow within and among them, suggesting that rivers and streams are important for seed dispersal [89, 90]. Indeed, river and stream habitats have long been recognized as corridors for riparian plants [35, 38, 91]. The ‘unidirectional dispersal hypothesis’ [36] predicts that downstream accumulation of genetic diversity results from the movement of seeds and propagules from upstream to downstream populations due to a continuous influx of alleles. Some studies have demonstrated such a relationship between the position of plant populations along the longitudinal course of a river and the degree of genetic diversity within these populations [32, 33, 35, 92]. In contrast, the present study based on both cpDNA and ISSR data showed that the genetic diversity in local populations of *C. coenobialis* at downstream locations was significantly lower than upstream, whether in the linear streamside local populations across DH (*H*: 0.091 vs 0.136) or in the dendritic streamside local populations across NK (*H*: 0.079 vs 0.112, 0.110). This implies that downstream local populations do not accumulate genetic diversity, which we attribute to the absence of movement of seeds and propagules from upstream to downstream local populations. This was confirmed by a lack of accumulation of particular cpDNA haplotypes in downstream local populations (e.g. DH: local population-FST; NK: local population-SH), whether in the linear streamside local populations across DH (haplotypes H7, H8) or in the dendritic streamside local populations across NK (h6, h7, h11, h12). In addition, the high degree of differentiation and low level of gene flow (*N*m) among local populations of *C. coenobialis* also suggests that seed dispersal among streamside local populations has been restricted across both DH and NK. The limited seed dispersal in *C. coenobialis* is apparent from the significant positive autocorrelation of spatial genetic structure being detected only at 0.20—0.92 m. Our field observations showed that the small seeds of *C. coenobialis*, which adhere to the axial placentation in an unilocular capsule that opens via a large oval slit in the upper part (Fig. S1), can be dispersed by rain splash [66, 67], like other species of the same genus [93, 94]. However, the stream terrain in mountainous areas is complex, characterized by irregular topography and geographical obstacles, which prevent the minute *C. coenobialis* seeds being propagated over long distances by water along the stream. This therefore reduces gene flow and increases genetic divergence among plant populations. Moreover, the plant hangs on rock walls near streams in monsoon forests, which means that upstream seeds are less likely to deposit among downstream local populations. We suggest that, recently at least, stream basins and the direction of water flow are not important for seed dispersal of *C. coenobialis* among local populations; in other words, such streams are not acting as corridors for propagation. However, the ancestral haplotypes shared among local populations across both DH (H1, H2, and H6) and NK (h1, h3, and h5) indicate that the connectivity of selfing *C*. *coenobialis* local populations in the two mountain areas studied could be attributed to historical gene flows, which occurred between the Holocene and Pleistocene. Further study is needed to investigate these hypotheses in more detail.

## Materials and methods

### Species, study sites and sampling design

*Caulokaempferia coenobialis* (Hance) K. Larsen (Fig. S1) is a deciduous perennial nonclonal herb of up to 50 cm in height that is endemic in the Guangdong, Guangxi, and Yunnan provinces of south China, where it grows on rock walls usually along streams in humid monsoon forests and is over the streams ca. 1–4 m. The plant flowers in May to August and is self-fertilizing by virtue of pollen sliding to the stigma through an oily emulsion [64–66]. Capsules mature within 22 days, and burst open or dehisce [64–66]. Seeds are small (ca. 1.65 ± 0.06 mm × 0.59 ± 0.07 mm) and ellipsoid-ovoid, without aril and appendages to aid wind dispersal, and dispersed by rain splash, and then germinate and develop into seedlings that develop rhizomes before the plants die back between September and November [64, 66, 67].

To investigated the patterns of genetic diversity and structure within and between populations in subtropical monsoon forest at a fine scale, two metapopulations with different streamside local population systems were studied in the Dinghu Mountain National Nature Reserve (DH: 23°09′21"-23°11′30" N, 112°30′39"-112°33′41" E, alt. 1000.3 m) and Nankun Mountain Provincial Nature Reserve (NK: 23°35′14"-23°43′05" N, 113°48′41"-113°56′32" E, alt. 1210 m), both in Guangdong, but separated by nearly 160 km (Table 1, Fig. 2A). The DH metapopulation consists of four streamside local populations along a linear perennial stream (pop. JLS, TXL, RZPB, and FST, Fig. 2 B1), which are naturally separated by 300 m—2.3 km (average ca. 583 m), with little interference from human activities. The NK metapopulation consists of nine local populations, which are split into two subsets, the West-North area (NK-I) and the East-South area (NK-II), by the G355 National Road along a valley in Nankun Mountain (Fig. 2 B2), and are greatly disturbed by human activities. The subset in the West-North area consists of five streamside local populations along a dendritic perennial stream (pop. TTD, TYSZ, GYT, XXPB, and SH), which are separated by 1.2 km—5.9 km (average ca. 2.1 km) of farmland, village, road and mountain forest. The subset in the East-South area consist of four streamside (pop. YXT, YTH and SLCX) or forested (pop. SLCL) local populations in a complex transverse hydrological system, which are isolated by average 333 m—2.2 km (average ca. 541 m) of mountain forest, agricultural land, village, road and holiday villa. In each local population, we sampled 20 adult individuals (except pop. FST: 10 individuals at least 0.5 m apart) throughout the area of distribution of each local population; there are ca. 500 to 10,000 mature individuals per local population (Table 1). The straight-line distance between individuals was also estimated directly on the basis of the site coordinates to test the spatial autocorrelation coefficient (r) within local populations. Leaf tissue samples were stored in silica gel for DNA analysis. The formal identification of plant materials was performed by the correspondence author, Professor Ying-Qiang Wang. The herbarium vouchers of *C. coenobialis* (WYQ-HHDBJ-5 and LGH-HHDBJ-81) were deposited in the Herbarium of School of Life Science, South China Normal University (SN). The field work permits were obtained from the Dinghu Mountain National Nature Reserve Administration and the Nankun Mountain Provincial Nature Reserve Administration. The sample collection work and molecular experiments complied with local legislation, national and international guidelines, and did not involve protected species. We also abide by the Convention on the Trade in Endangered Species of Wild Fauna and Flora.

### DNA extraction, chloroplast DNA sequencing and ISSR fragment analysis

Total genomic DNA was extracted from 0.2 to 0.5 g dried leaves using a modified 2% CTAB protocol [95]. The quality and concentration of the extracted DNA were estimated on a 0.8% agarose gel and a Nano-100 spectrophotometer (Allsheng, China).

We screened eight non-coding plastid DNA regions that have revealed substantial levels of polymorphism based on chloroplast genome sequence of *Zingiber spectabile* [96] and then selected the two most variable cpDNA regions for analysis, including one intergenic spacer region (psbJ–petA) and one gene intron (trnL intron). Reactions were performed in a total volume of 20 μL containing 2.0 μL 10 × PCR buffer, 1.5 mM MgCl_2_, 0.2 mM dNTPs, 0.25 μM forward primer, 0.25 μM reverse primer, 0.75 units Taq polymerase, 37.5 ng template DNA and double-distilled water. Polymerase chain reactions (PCRs) were conducted on a Bio-Rad T100™ Thermal Cycle (Bio-Rad, Singapore) under the following conditions: initial denaturation at 94 °C for 5 min, followed by 39 cycles of 45 s at 94 °C, 45 s at a primer-specific annealing temperature, extension for 90 s at 72 °C, and a 10-min final extension step at 72 °C. The PCR products were sequenced with an ABI 3730XL automated sequencer (Applied Biosystems, Foster City, CA).

For ISSR analysis, ten polymorphic primers (807, 808, 810, 835, 836, 840, 841, 847, 859, and 887) were selected from 100 ISSR primers obtained from the University of British Columbia. Reactions were performed in a total volume of 20 μL containing 2.0 μL 10 × PCR buffer, 1.5 mM MgCl_2_, 0.2 mM dNTPs, 1.0 μM primer, 1.5 units Taq polymerase, 50 ng template DNA and double-distilled water. ISSR PCR conditions were consistent with cpDNA PCR conditions. Amplification products were electrophoretically separated in 1.8% agarose gels, together with a 100 bp ladder as a size marker, and visualized on a UV transilluminator (Bio-Rad Gel Doc XR + , America). All clear and reproducible amplified fragments were scored as presence (1) or absence (0) and converted into a binary data matrix. The annealing temperatures for cpDNA and ISSR primers are given in Table 2.

### Data analysis

#### Genetic diversity, genetic differentiation and gene flow

For the cpDNA dataset, sequence data were aligned using MEGA X [97] and were manually adjusted where necessary. Contiguous indels were treated as single mutation events and coded as substitutions (A or T) [98]. The number of haplotypes, haplotype diversity (*h*), nucleotide diversity (π) and gene flow (*N*m) were calculated using DNASP v5.1 [99]. The permutation test implemented in PERMUT was employed to compare parameters of local population differentiation with ordered alleles (*F*_ST_) based on 1000 random permutations [100]. Pairwise estimates of uncorrected sequence divergence (Dxy, Kimura 2-parameter model) among local populations were calculated in MEGA X. Standard deviations were determined using 1000 bootstrap replicates.

For the ISSR dataset, Nei’s gene diversity (*H*) [101], Shannon’s index (*I*) [102], percentage of polymorphic loci (PPL), gene differentiation (*G*_ST_) [103], and gene flow were estimated using the program POPGENE version 1.32 [104], assuming complete selfing within local populations (*F*is = 1) because *C. coenobialis* plants are self-fertilization [64, 65]. Pairwise estimates of Nei’s genetic distance among local populations were calculated in POPGENE version 1.32.

In order to quantify the variation in cpDNA sequences and ISSR gene frequency among local populations, we performed analyses of molecular variance (AMOVA) in ARLEQUIN v3.1 [105] and GENALEX ver.6.5 [106] using *R*- and *Φ*-statistics, respectively. The significance of fixation indices was tested using 1,000 and 999 permutations, respectively [107].

#### Genetic structure and cluster analysis

For the cpDNA dataset, phylogenetic trees were constructed to reveal the relationships among haplotypes. Relationships for the identified haplotypes were reconstructed using maximum likelihood (ML) and Bayesian inference (BI). Two individuals of *Pyrgophyllum yunnanense* and *Kaempferia rotunda* were used as the outgroup. ML analysis was carried out using MEGA X and the appropriate model (T92 + G + I) of DNA substitution was determined using the Akaike information criterion (AIC) as the selection criterion. The ModelFinder in PhyloSuite v.1.1.15 [108] was applied to find the F81 + F + I model for BI methods based on the AIC. BI analysis was conducted in MrBayes 3.2.6 [109] with the following settings: 1,000,000 metropolis-coupled Markov chain Monte Carlo (MCMC) generations, sample frequency of 1,000 and burn-in parameter set at 2,500. Then, as no fossil records are available to calibrate the intergenic spacer substitution rate for genus *Caulokaempferia*, branch lengths of the clock-constrained ML tree were transformed into absolute time by assuming the substitution rates of these spacers to be 1.2–1.7 × 10^–9^ substitutions per site per year (s/s/y) in MEGA X. This is a rough estimate of the substitution rate in non-coding chloroplast regions of seed plants, which can be used to estimate the divergence time of taxa without fossil records in the same study area (e.g., *Sinopodophyllum hexandrum*, [110]; *Rosa soulieana*, [111]).

For the ISSR datasets, a Bayesian cluster was implemented using STRUCTURE version 2.2 [112]. Five independent runs were performed for each K, from K = 1 to 4 for the DH metapopulation and from K = 1 to 9 for the NK metapopulation. All runs were performed with the admixture model, with burn-in and run lengths of 100,000 and 1,000,000 iterations, respectively. The optimal number of clusters (ΔK) was determined following the guidelines of [112] and the recommendations of Evanno et al. [113]. Individual assignment coefficients (*q*) for each genetic cluster were then averaged using Clumpp software [114] to correct for any discrepancies between runs. To further test the genetic relationship among individuals, we constructed a neighbor-joining (NJ) tree based on Nei's genetic distance using the program DARwin 6.0.9 [115] with 1,000 re-samples for bootstrap support, an unweighted pair-group method arithmetic mean (UPGMA) dendrogram based on Nei's similarity coefficient using NTSYS 2.1 [116], and principal coordinate analysis (PCoA) based on Nei’s genetic distance in GenAlEx ver. 6.5.

#### Analysis of spatial genetic structure

To test the relationship of geographical distance and genetic structure between local populations on a microgeographic scale, a Mantel test was performed in GENALEX ver.6.5 for ISSR data and ARLEQUIN v3.1 for cpDNA data, respectively. To detect spatial genetic structure (SGS) within a local population, the genetic relatedness of individuals relative to their spatial position within the TXL local population in DH was analyzed by spatial autocorrelation analyses in GenAlEx. The even sample classes were chosen because this was particularly useful for reducing noisy confidence limits when sample sizes were very uneven. Two-tailed probability values were calculated and bootstrap resampling was performed 999 times.

## Conclusion

Using polymorphic ISSR and cpDNA loci, we studied the effect at a microgeographic scale of different stream systems (a linear stream, a dendritic stream, and complex transverse hydrological system) in subtropical monsoon forest on the genetic structure and connectivity of a riparian self-fertilizing herb *Caulokaempferia coenobialis* populations, which does not use anemochory or zoochory for seed dispersal, across Dinghu Mountain and Nankun Mountain. Such studies could contribute to an improved understanding of the effect of rivers or streams on population genetic diversity and structure in riparian plants. Our results show that streams are not acting as corridors for dispersal of *C. coenobialis* and the connectivity of local populations can be attributed to historical gene flows, resulting in a lack of spatial genetic structure, despite self-fertilization. Selfing *C. coenobialis* can maintain high genetic diversity through genetic differentiation, which is intensified by local adaptation and neutral mutation and/or genetic drift in local populations at a microgeographic scale.

## Supplementary Information

**Additional file 1:Fig. S1.***Caulokaempferia coenobialis*. a: plant, flower and habitat; b: seeds adhered to the axial placentation in an unilocular capsule opening by a large oval slit; c: seeds dispersed by rain splash. **Fig. S2**. Line graph of genetic cluster (K) vs. Delta K for metapopulations DH (A) and NK (B). **Fig. S3**. UPGMA dendrogram based on Nei's genetic identity for individuals of *Caulokaempferia coenobialis* in metapopulations DH (A) and NK (B). **Table S1**. Inter Simple Sequence Repeat (ISSR) data for *Caulokaempferia coenobialis*.

## Data Availability

The two plastomic data analyzed in this study are deposited into the NCBI database (https://www.ncbi.nlm.nih.gov/nucleotide/) with accession numbers: MW849477-MW849520; Inter Simple Sequence Repeat (ISSR) data for *Caulokaempferia coenobialis* is available as Supplementary Information.
