# Supplementary material for: Population genetic structure and connectivity of a riparian selfing herb Caulokaempferia coenobialis at a fine-scale geographic level in subtropical monsoon forest
Source: BMC Plant Biol. 2021 Jul 8;21:329. doi: 10.1186/s12870-021-03101-7 (PMC8265151; doi:10.1186/s12870-021-03101-7)
Supplement: Supplementary file 1 — Additional file 1:Fig. S1.Caulokaempferia coenobialis. a: plant, flower and habitat; b: seeds adhered to the axial placentation in an unilocular capsule opening by a large oval slit; c: seeds dispersed by rain splash. Fig. S2. Line graph of genetic cluster (K) vs. Delta K for metapopulations DH (A) and NK (B). Fig. S3. UPGMA dendrogram based on Nei's genetic identity for individuals of Caulokaempferia coenobialis in metapopulations DH (A) and NK (B). Table S1. Inter Simple Sequence Repeat (ISSR) data for Caulokaempferia coenobialis. [file 12870_2021_3101_MOESM1_ESM.zip › FQ-Supplementary material.docx]

**Population genetic structure and connectivity of a riparian selfing herb** ***Caulokaempferia coenobialis*** **at a fine-scale geographic level in subtropical monsoon forest**

Qiong Fu^1, †^, Jie Deng^1, †^, Min Chen^1, †^, Yan Zhong^1^, Guo-Hui Lu^1^, Ying-Qiang Wang^1, 2^**^*^**

1 Guangdong Provincial Key Laboratory of Biotechnology for Plant Development, School of Life Sciences, South China Normal University, Guangzhou, China

2 Guangzhou Key Laboratory of Subtropical Biodiversity and Biomonitoring, School of Life Sciences, South China Normal University, Guangzhou, China


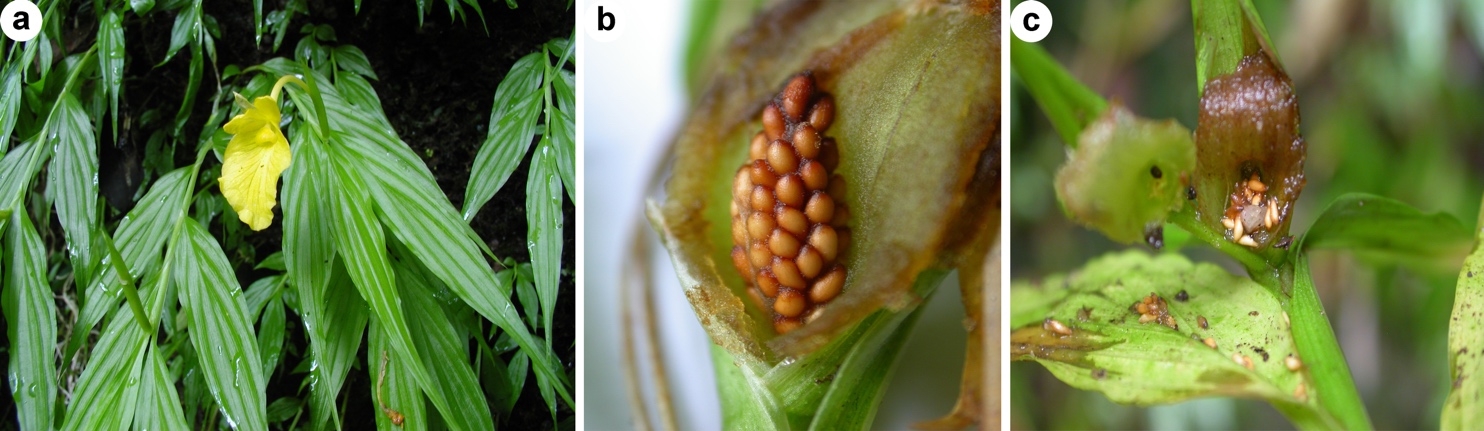


Fig. S1 *Caulokaempferia coenobialis*. a: plant, flower and habitat; b: seeds adhered to the axial placentation in an unilocular capsule opening by a large oval slit; c: seeds dispersed by rain splash.

(A)


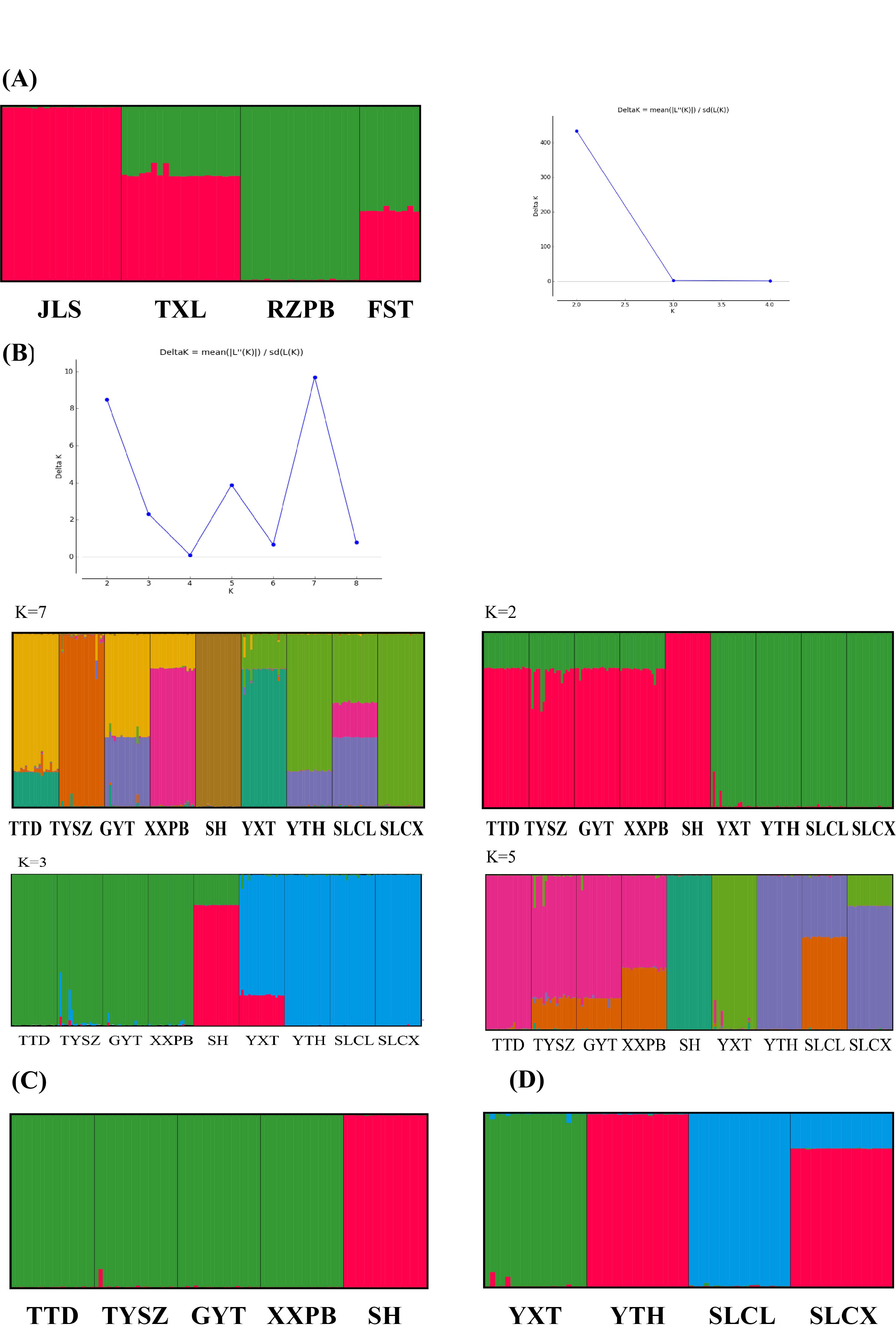


(B)


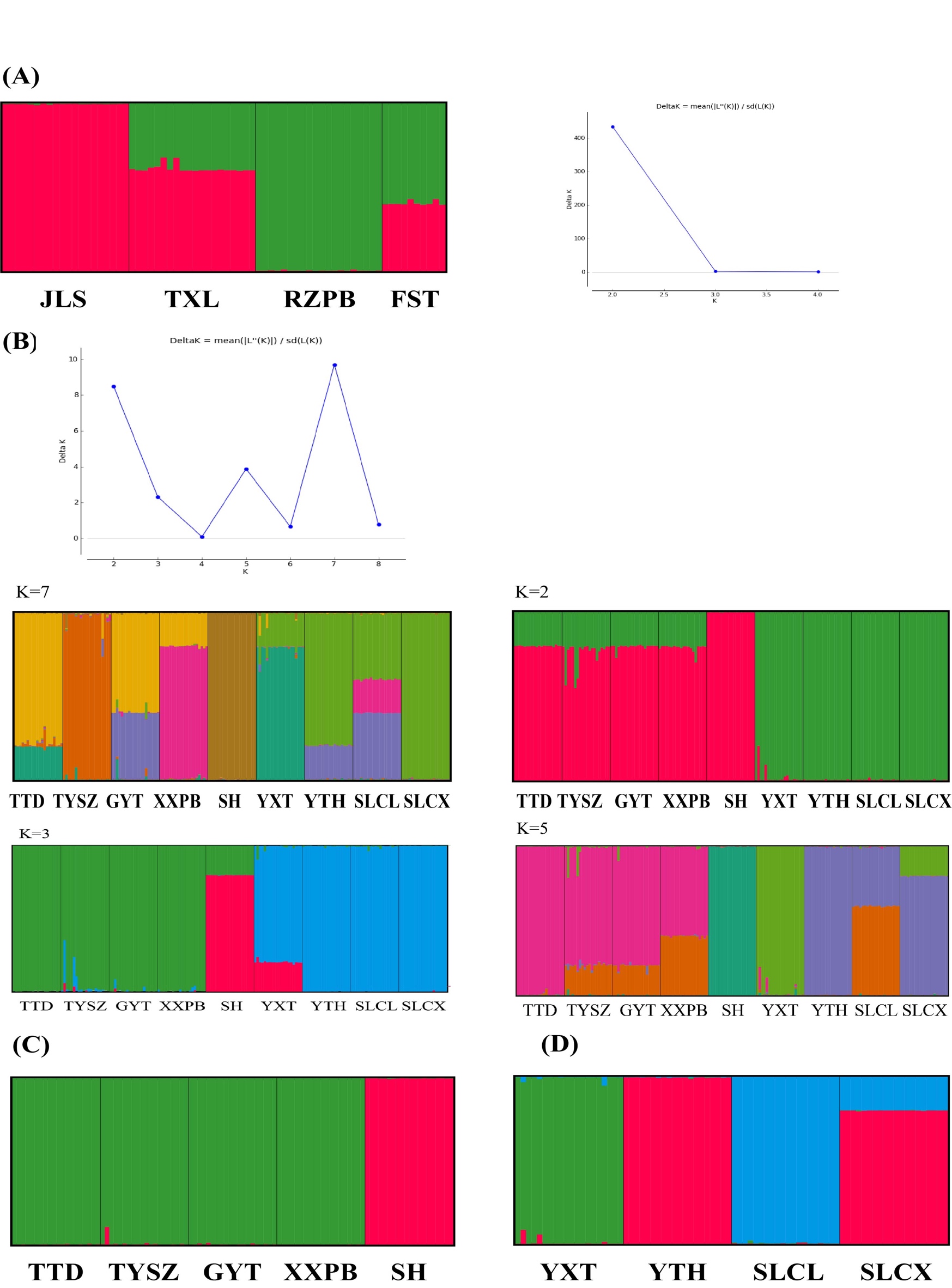


Fig. S2 Line graph of genetic cluster (K) vs. Delta K for metapopulations DH (A) and NK (B).


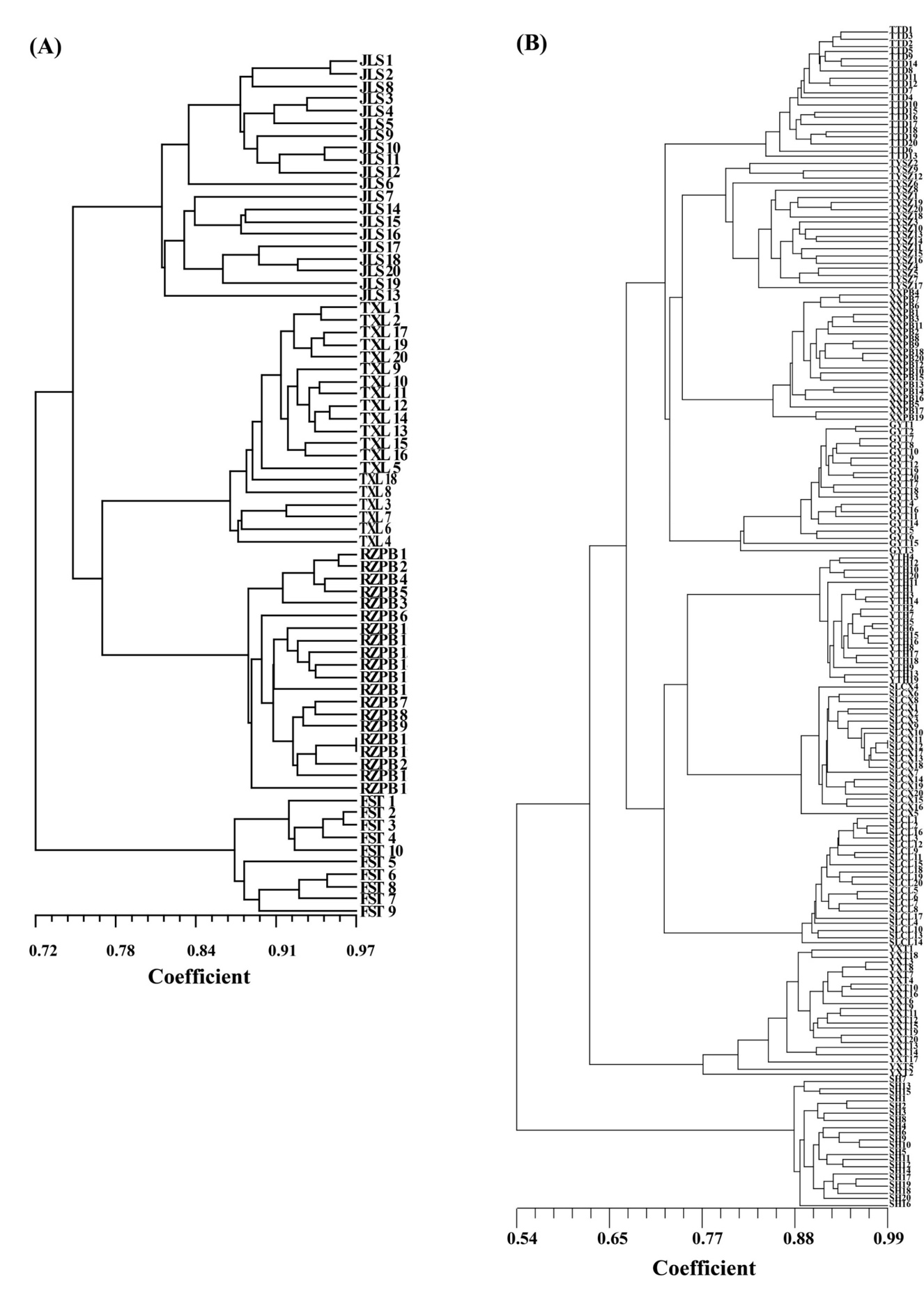


Fig. S3 UPGMA dendrogram based on Nei's genetic identity for individuals of *Caulokaempferia coenobialis* in metapopulations DH (A) and NK (B).
